# Occupational health burden of handloom workers: a systematic review and meta-analysis

**DOI:** 10.3389/fpubh.2026.1828924

**Published:** 2026-07-22

**Authors:** Chandan Kumar Swain, Himanshu Sekhar Rout, Simpul Behera, Arabinda Mishra, Rabi N. Patra, Mitali Chinara, Umakanta Sahoo, Anjali Dash

**Affiliations:** 1 Department of Analytical & Applied Economics, Utkal University, Bhubaneswar, India; 2 Population Research Centre, Utkal University, Bhubaneswar, India; 3 Development & Environment Futures Trust, Bhubaneswar, India; 4 Department of Statistics, Sambalpur University, Sambalpur, India; 5 Department of Economics, Panchayata College, Bargarh, India

**Keywords:** burden of disease, handloom weaver, incidence, occupational hazards, prevalence, risk factors

## Abstract

**Background:**

Handloom is a traditional textile production sector, and the handloom workers work in risky conditions that may adversely affect their health and wellbeing. Although occupational health risks are highly significant issues, limited comprehensive studies exist on the overall prevalence of diseases among handloom workers. A systematic review, along with a bibliometric and meta-analysis of existing literature on health risks associated with hazardous work environments in the handloom industry, is presented in this paper.

**Methods:**

Data were extracted from Scopus, Web of Science, and PubMed databases. After full-text screening, 47 eligible articles were shortlisted for this paper. Bibliometric analysis was done by using the biblioshiny package in RStudio. For the meta-analysis, a random-effects model was used with 95 percent confident interval.

**Results:**

The annual growth rate of publications in this field was found to be 1.55 percent between 1980 and 2024, indicating a gradual increase in research interest. Thematic trends in literature reveal a shift from economic history and industrial transformation to public health issues such as musculoskeletal disorders and cross-sectional health studies. The meta-analysis shows a higher relative risk of 1.24 (*P* = 0.16) for musculoskeletal disorders among handloom workers compared to control groups which was not significant. Moreover, the overall disease prevalence among handloom workers shows a relative risk of 1.03 (*P* = 0.78) than the control group. This statement shows that no significant difference in the overall prevalence of diseases among handloom workers compared to the control groups. The other occupational related health issues identified among handloom workers include eye strains, respiratory illnesses, digestive disorders, and visual impairments.

**Conclusions:**

These findings underscore the need for targeted occupational health policies and interventions to mitigate health risks among handloom workers.

## 1 Background

Occupational health problems refer to the health issues of workers that arise due to an unhealthy working environment. Workers' exposure to occupational risk, as measured by the summary exposure values (SEVs) per 100 people, has remained more or less static at the global level over the last three decades. The SEVs attributable to occupational risk rose from 3.58 in 1990 to 3.61 in 2021. Despite the almost unchanged scenario of the SEVs due to occupational risk, the trends of death per 100,000 people (death rate), occurring at the global level, have been declining, e.g., the death rate due to occupational health risk has fallen from 24.15 in 1990 to 18.19 in 2021, or by 24.25 percent. An additional indicator of the health status of workers is the disability-adjusted life years (DALY), a method used to compute a standardized unit of measure of health by combining morbidity and mortality. One DALY represents 1 year's loss of healthy life. If we assess the loss of health from occupational hazards by disease-wise, we find a higher loss of healthy life years due to transport injuries (212.46 DALY per 100,000 people), followed by musculoskeletal diseases (MSDs) (197.29 DALY per 100,000 people), unintentional injuries (191.26 DALY per 100,000 people), chronic respiratory diseases (187.68 DALY per 100,000 people), sense organ diseases (99.44 DALY per 100,000 people), and neoplasms (90.34 DALY per 100,000 people) in 2021 at the global level (1).

Handloom is an informal cottage industry. It is also a household-based industry, dominant in rural areas, labor-intensive, and mostly dependent on the female labor force. The working environment of the handloom sector is characterized by long hours of working, repetitive work (2–4), awkward and static posture (5–7), exposure to cotton dust (8) and vibration, a need for concentration (9), and a lack of noise control at the workplace (10). These occupational risks in the workplace led to occupational-related health problems like musculoskeletal disorders (11–16), respiratory problems (8), eye and ear problems (9), and injuries (17) among weavers. Due to occupation-related health problems in the handloom sector, the United Nations Sustainable Development Goals 3 and 8 (18), emphasizing health for all and productive employment and decent work for all, respectively, could not be achieved.

There is a lack of studies on bibliometric analysis of the health problems of handloom workers as of this date. Studies on the subject have focused mostly on specific diseases among handloom workers. Another gap is that no study has attempted a meta-analysis of the occupational health problems among handloom workers. Therefore, the present study has focused on the bibliometric analysis of the occupational health burden among handloom workers. Moreover, an attempt has been made in this study to perform a meta-analysis of the occupational health issues of handloom workers and a narrative review related to disease-specific occupational hazards and their risk factors among them.

## 2 Methodology

### 2.1 Data

**Figure 1:**
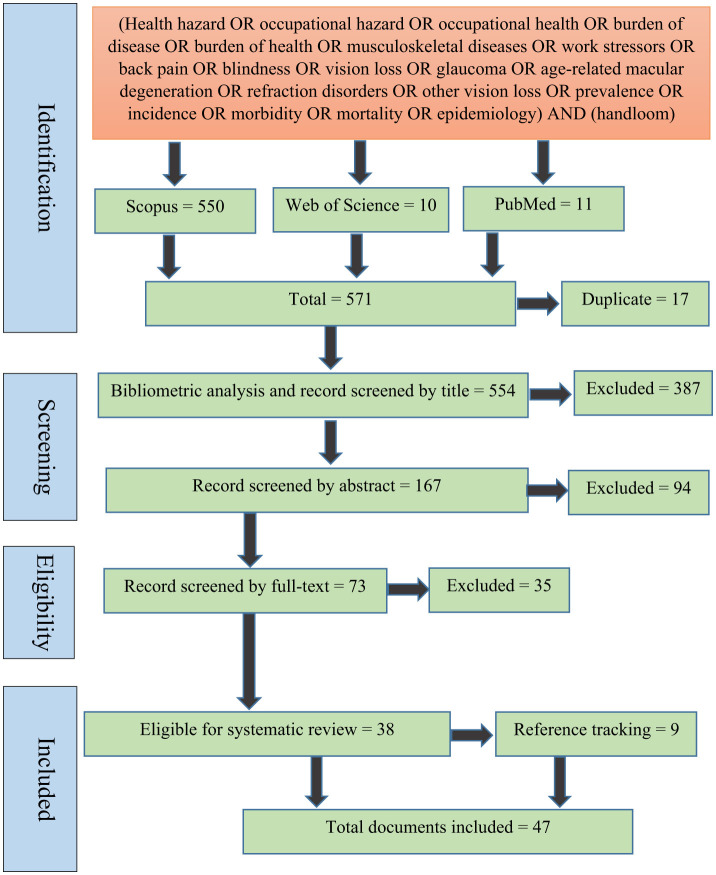
PRISMA flow chart. Source: Authors' prepared. Preferred reporting items for systematic reviews and meta-analyses.

The present study utilizes three major databases, such as Scopus, Web of Science, and PubMed. To conduct the systematic review and meta-analysis, we have followed the Preferred Reporting Items for Systematic Reviews and Meta-Analyses (PRISMA) guidelines (19). The details of the PRISMA flow chart are represented in Figure 1.

#### 2.1.1 Search strategy

This study included all publications up to February 2025, without selecting any filter option. Considering all fields for searching documents by advanced search option, the following keywords were identified: (health hazard OR occupational hazard OR occupational health OR burden of disease OR burden of health OR musculoskeletal diseases OR work stressors OR back pain OR blindness OR vision loss OR glaucoma OR age-related macular degeneration OR refraction disorders OR other vision loss OR prevalence OR incidence OR morbidity OR mortality OR epidemiology) AND (handloom). In addition, reference tracking was done for search documents.

#### 2.1.2 Inclusion and exclusion criteria

The present study included only the health aspects of handloom workers, such as incidence, prevalence, risk factors, and determinants of diseases. It also considers the comparative studies between the handloom workers and control groups. The control groups were worker other than handloom workers. This study excluded studies that could not focus on the health aspects of handloom workers. Moreover, publications that focused on health but did not focus on handloom workers, were also excluded from this study.

### 2.2 Methods

Bibliometric analysis, narrative review, and meta-analysis were used for the present study. Bibliometric analysis was done by using the biblioshiny package in RStudio. For meta-analysis, Stata 17 was used.

For the systematic review and meta-analysis Newcastle–Ottawa Scale (NOS) tool is preferable than NIH tool (20). So, the present study used the NOS to evaluate the quality of literature and risk of bias involved in its methodology. Total of the nine stars has been given to the three aspects of studies such as selection, comparability and outcome. The maximum 4 points are assigned to selection, 2 points for comparability and 3 points for outcome of study. The details of the items coming under the three aspects were explained in the online materials (21). To find out the overall score of the NOS, first the geometric mean of each aspect was estimated. If one item is zero, geometric mean cannot be estimated hence 0.01 added to each item of each aspect. By using the score of the three aspects, the weighted geometric mean estimate which given the overall score of study. The weightage of each aspect equal to its theoretical maximum score.

In addition, the classification of studies by its each aspect and overall score by the three categories such as poor, fair, and good. The overall score of each study was varied between 0.01 and 1.18. The details of the quality classification of study are provided in Tables 1 and 2.

**Table 1:** Classification of studies by its aspects.

Classification of studies	Number of points		
	Selection	Comparability	Outcome
Good	3 or 4	2	3
Fair	2	1	2
Poor	0 or 1	0	0 or 1

Source: Authors' estimated.

**Table 2:** Classification of studies by its overall quality.

Classification of studies	Overall value of studies	
	Lower value	Upper value
Poor	0.00	0.65
Fair	0.66	0.95
Good	0.96	1.18

Source: Authors estimated.

When outcomes were binary, the relative risk (RR) estimated happens between the two groups. In the present study binary outcomes depend on the number of success and failure cases. We compared the risk of the prevalence of the diseases between the handloom workers (treated group) and the other workers (control group). For both groups, the number of success cases was the number of prevalences of disease, and the number of failure cases was the number of persons living without the prevalence of diseases. RR was estimated by using Stata version 17.

There are two types of regression model used, i.e., fixed effect and random effect. A fixed effect is used when no significant heterogeneity is found across studies, while a random effect is used when significant heterogeneity is found. In the present study, initially we have used the fixed effect and found the significant heterogeneity represented by I^2^ and its corresponding *P-value* at the 5 per cent level of significance. Therefore, we have changed our model from a fixed effect to a random effect. Due to significant heterogeneity across studies, it leads to a wider confidence interval in random effects than in fixed effects (22). In addition, to know the effect size, we have reported the exponentiated effect sizes of the log risk ratio, which give the relative risk of the prevalence of diseases between the control group and the treated group.

There are three possible interpretations of relative risk: RR = 1, RR > 1, and RR < 1. When RR equals 1, there is no difference in disease risk between the control and treatment groups. When RR is greater than 1, the treatment group has a higher risk of disease compared to the control group. Conversely, when RR is less than 1, the treatment group has a lower risk of disease compared to the control group.

To know the reasons for the presence of heterogeneity in meta-analysis, used of the leave-one-out sensitivity analysis which helps for finding out individual study's effect in pooled effect size. To assess publication bias, both funnel plots and Egger's test were employed. The funnel plot provides a graphical representation of publication bias in the other hand Egger's test offers a statistical method for its detection. Publication bias is also referred to as the small-study effect. Egger's test is considered more robust than the funnel plot because it relies on objective statistical assessment whereas interpretation of funnel plots can be subjective. A symmetrical distribution of studies in the funnel plot suggests a low likelihood of publication bias, whereas asymmetry indicates a higher likelihood. The null hypothesis of Egger's test states that there is no publication bias.

## 3 Results

### 3.1 Risk of bias assessment

**Figure 2:**
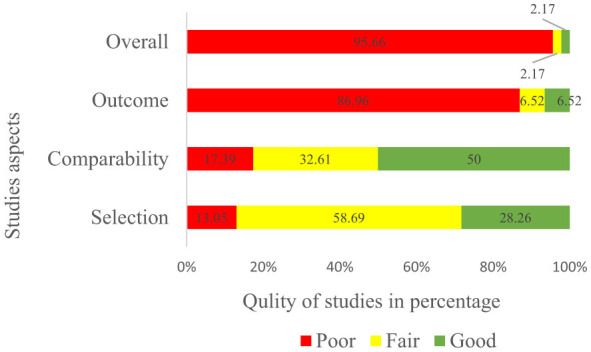
Quality of studies. Source: Authors estimated from review of literature.

The risk of bias assessment reflects the methodological quality of the included studies. There is a positive relationship between the NOS score and quality of the study with higher scores indicating better quality. The present study found that most studies were classified under the fair category (58.69%) followed by the good category (28.26%) in the selection domain. In the comparability domain, half of the studies were categorized as good (50%) followed by fair (32.61%). However, in terms of the overall score 95.66 per cent of studies were classified as poor. This was primarily due to 86.96 per cent of studies falling into the poor category in the outcome domain (Figure 2).

Both cohort and cross-sectional studies were included in this analysis, with the majority being cross-sectional in design. The NOS, which is primarily designed for cohort studies, was used to assess the risk of bias. As a result, many cross-sectional studies failed to receive points for the follow-up criterion within the outcome domain. Therefore, despite relatively better performance in the selection and comparability domains, the overall quality of most studies was rated as poor.

### 3.2 Narrative review

According to the inclusion criteria of the reviewed literature, 47 studies were eligible for inclusion in the present study for a narrative review. Out of 47 studies included in the present study, 43 studies (91.49%) were focused on occupational diseases among handloom workers and the remaining 4 studies (8.51%) were concentrated on occupational risk factors like ergonomic risks [2 studies (4.25%)], postural assessment [1 study (2.13%)], and health effects of handloom dyeing [1 study (2.13%)]. This has been presented in Table 3. Additionally, based on the classification of occupational diseases among handloom workers, it was found that 33 studies (70.21%) were focused on musculoskeletal diseases, 5 studies (12.64%) on multiple diseases, 2 studies (4.25%) on the nervous system, 1 study (2.13%) on the circulatory system, 1 study (2.13%) on the visual system, and 1 study (2.13%) on the respiratory system.

**Table 3:** Features of the reviewed literature on the occupational hazards in the handloom sector.

References	Country	Types of data	Sample size			Disease or risk factors	Comparison group
			Total	Male	Female		
Tiwari et al. (8)	India	Primary^1^	638	490	148	Respiratory system	Occupation other than handloom
Gangopadhyay et al. (16)	India	Primary^1^	125	125	NI	Musculoskeletal disorder	Meat cutters, typists, tailors, and visual display terminal operators
Choobineh et al. (24)	Iran	Primary^1^	1,439	1,439	29	Musculoskeletal disorder	General people
Choobineh et al. (15)	Iran	Primary^2^	1,439	1,439	29	Musculoskeletal disorder	General people
Nag et al. (10)	India	Primary^1^	516	253	263	Musculoskeletal disorder	Power loom weavers
Motamedzade and Moghimbeigi (29)	Iran	Primary^1^	626	NI	626	Musculoskeletal disorder	No comparison
Nazari et al. (38)	Iran	Primary^1^	200	117	83	Musculoskeletal disorder	No comparison
Wani and Jaiswal (40)	India	Primary^1^	766	NA	NA	Multiple diseases	No comparison
Marmamula et al. (9)	India	Primary^1^	2,828	1,283	1,565	Visual system	No comparison
Pandit et al. (39)	India	Primary^1^	50	NA	50	Musculoskeletal disorders	No comparison
Durlov et al. (36)	India	Primary^1^	175	NA	NA	Musculoskeletal disorders	No comparison
Chaman et al. (37)	Iran	Primary^1^	546	NI	546	Musculoskeletal disorders	No comparison
Chantaramanee et al. (46)	Thailand	Primary^1^	105	NA	NA	Ergonomic risks	No comparison
Wani et al. (41)	India	Primary^1^	231	71	160	Musculoskeletal disorders	No comparison
Karimi et al. (14)	Iran	Primary^1^	862	224	638	Musculoskeletal disorders	No comparison
Mahdavi et al. (13)	Iran	Primary^1^	12	3	9	Musculoskeletal disorders	No comparison
Nag et al. (10)	India	Primary^1^	1,122	NA	NA	Multiple diseases	Power loom, transport, tobacco processors, fish processors, construction workers
Rahman et al. (31)	Bangladesh	Primary^1^	450	NA	NA	Musculoskeletal disorder	No comparison
Parida (5)	India	Primary^1^	NA	NA	NA	Musculoskeletal disorders	No comparison
Singh et al. (44)	India	Primary^1^	170	NI	170	Nervous system	Block printer, jewelry worker, and office worker
Bori and Bhattacharyya (25)	India	Primary^1^	60	NI	60	Postural assessment	No comparison
Lakshmi et al. (51)	India	Primary^1^	NA	NA	NA	Musculoskeletal disorder	No comparison
Ramdan et al. (57)	Indonesia	Primary^1^	40	NI	40	Musculoskeletal disorder	No comparison
Satheeshkumar and Krishnakumar (32)	India	Primary^1^	361	274	87	Musculoskeletal disorder	No comparison
Singh and Singh (58)	India	Primary and secondary^1^	NA	NA	NA	Multiple diseases	No comparison
Suryati and Nggarang (12)	Indonesia	Primary^1^	40	NA	NA	Musculoskeletal disorder	No comparison
Inovero et al. (42)	Philippines	Primary^1^	20	NI	20	Circulatory system	No comparison
Bori and Bhattacharyya (25)	India	Primary^1^	180	NI	180	Multiple diseases	No comparison
Mallapiang et al. (28)	Indonesia	Primary^1^	124	NA	NA	Musculoskeletal disorder	No comparison
Ramdan and Candra (48)	Indonesia	Primary^2^	40	NI	40	Musculoskeletal disorder	No comparison
Ruliati et al. (11)	Indonesia	Primary^1^	47	NI	47	Musculoskeletal disorder	No comparison
Sawadogo et al. (59)	Burkina Faso	Primary^1^	213	NA	NA	Musculoskeletal disorder	No comparison
Singh et al. (45)	India	Primary^1^	120	NI	120	Nervous system	Hand block printing workers and imitation jewelry workers
Siddiqui et al. (26)	India	Primary^1^	364	NA	NA	Musculoskeletal disorder	Power loom weavers
Muthukumar et al. (33)	India	Primary^1^	206	114	92	Musculoskeletal disorder	No comparison
Haftu et al. (23)	Ethiopia	Primary^1^	420	419	1	Musculoskeletal disorder	No comparison
Islam et al. (47)	Bangladesh	Primary^1^	NA	NA	NA	Health effects of handloom dyeing	NA
Kathirvelu et al. (49)	India	Primary^1^	183	141	42	Musculoskeletal disorder	No comparison
Yogeshwaran and Suresh (30)	India	Primary^1^	30	30	NI	Musculoskeletal disorder	No comparison
Boonkhao et al. (43)	Thailand	Primary^1^	34	28	6	Ergonomic risk	No comparison
Kabore and Schepens (7)	Burkina Faso	Primary^1^	257	NI	257	Musculoskeletal disorder	No comparison
Terfe et al. (2)	Ethiopia	Primary^1^	660	591	69	Musculoskeletal disorder	No comparison
Khan and Qayum (27)	India	Primary^1^	22	13	9	Multiple diseases	No comparison
Sawadogo et al. (50)	Burkina Faso	Primary^1^	12	NI	12	Musculoskeletal disorder	No comparison
Stalin et al. (3)	India	Primary^1^	121	85	36	Musculoskeletal disorder	No comparison
Zinabu et al. (4)	Ethiopia	Primary^1^	424	209	205	Musculoskeletal disorder	No comparison
Meher et al. (35)	India	Primary^1^	435	NA	NA	Musculoskeletal disorders	No comparison

Source: Authors compiled from review of literature.

Super script 1 = cross-sectional study; Super script 2 = more than 1 round study; NI = not included in the study; NA = included in the study but detailed information not provided.

#### 3.2.1 Musculoskeletal diseases

The prevalence of MSDs varied across studies due to the variation in the working environment in the handloom industry. The prevalence of MSDs among handloom workers ranged from 46.9 percent in Ethiopia (23) to 100 percent in Iran (24), India (25–27), and Indonesia (28). A comparison of the prevalence of MSDs by gender reveals that there was a higher risk of occurrence among female workers compared to male workers (29). For males, MSDs ranged from 43 percent (30) to 100 percent (27), while for females, it ranged from 57 percent (30) to 100 percent (27). The lower value of the prevalence of MSDs for females was higher than males, which could be a true reflection of higher prevalence of MSDs among females than males. There was also variation in the prevalence of MSDs across different regions of the handloom workers' body (7, 24, 29–35). Most studies found that highest prevalence of MSDs was in the lower back (4, 5, 7, 32, 36, 37), followed by the neck (38, 39). Moreover, the highest prevalence for each musculoskeletal condition was reported in one study each for the hips (31), upper back (26), shoulder (23), and hands (25).

Age group-wise variation in the prevalence of MSDs has also been observed in the findings of reviewed research publications. Rahman et al. (31) found that all types of MSDs had the highest prevalence among the 46-years-and-above age group of weavers compared to those below 45 years. It has also been noticed that with the growing age, the prevalence of MSDs in different body regions was also rising, except for elbow, wrist, and knee pain. The lower prevalence of elbow, wrist, and knee pain was relatively high among weavers aged below 30 years in comparison to the 30–45-year-old weavers. Not only did the variation in the prevalence of MSDs across age groups vary, but it also varied by the class of weaving activity. Weaving activity can be classified into six categories such as yarn extraction, yarn pre-preparation, dyeing, weft yarn preparation, warp yarn preparation, and drafting of warp yarn. The top three musculoskeletal disease prevalence in body regions by type of weaving activity were knee (95%), wrist (90%), and ankle (85%) in yarn extraction; back (93.33%), knee (90%), and ankle (80%) in yarn pre-preparation; wrist (93.94%), shoulder (90.19%), and upper arm (87.88%) in dyeing; shoulder (95.12%), back (95.12%), and lower arm (87.80%) in weft yarn preparation; back (95%), knee (90%), and ankle (85%) in warp yarn preparation; and back (100%), shoulder (100%), and upper arm (90%) in drafting of warp yarn. Among all, the highest risk for the prevalence of MSDs was among the drafters of warp yarn in weaving (33).

Some studies also found seasonal variation in the prevalence of MSDs (40, 41). The four most seasonally prevalent diseases were finger pain, wrist pain, back pain, and joint pain. The findings show that autumn posed a higher risk for wrist and joint pain. The prevalence of wrist pain peaked in autumn at 29.68 percent, while it dropped to 6.25 percent in summer. Similarly, joint pain showed the highest prevalence in autumn (59.37%) and the lowest in summer (48.95%). Summer was considered a protective period for the weavers, with significant reduction in the prevalence of musculoskeletal diseases. It is worth mentioning that finger pain, wrist pain, and joint pain were all least prevalent during the summer months (40).

Wani et al. (41) explained the prevalence of musculoskeletal disease by finding an interaction between the age groups of handloom workers and seasons. A significant rise in the prevalence of wrist pain with age occurs during the monsoon. There was also a considerable variation in the prevalence of finger pain during the monsoon across age groups. A higher prevalence of finger pain was observed among the 31–40-year-old-age group (73.3%) during the monsoon, followed by the age groups of 41–50 years (71.4%), 10–20 years (54.8%), and 21–30 years (53.3%). The opposite case was observed for the prevalence of hand pain during winter. A higher prevalence of hand pain was found among young age groups (93.7% for 10–20 years and 61.5% for 21–30 years) compared to age groups 31–40 years (60%) and 41–50 years (56.2%).

#### 3.2.2 Circulatory system

The average blood pressure among weavers was 135/80 mmHg during their rest time. It indicates the prevalence of high systolic blood pressure among weavers. The blood pressure at the time of weaving will most likely be higher compared to the same during resting. It indicates a higher risk of prevalence of high blood pressure among weavers (42). Boonkhao et al. (43) reported a 20 percent prevalence of hypertension among weavers. Another indicator of the circulatory system is resting heart rate; the average beats per minute were 78 among weavers during rest, which indicates a normal heart rate. The average beats per minute were 78 among weavers. This study also shows a significant relationship between posture at the workplace and heart rate (42).

#### 3.3.3 Nervous system

Singh et al. (44) estimated the muscular strength among female handicraft and office workers and found that muscular strength was lower among female weavers than female office workers. Among the handicraft workers, a higher hand grip and muscular strength were noticed among weavers compared to jewelry workers. However, a lower hand grip strength was observed among weavers when compared to block printing workers. It was observed that the right hand of weavers consistently demonstrated greater strength compared to the left. Among the female weavers, the highest strength was recorded in hand grip, followed by palmer pinch grip, key pinch grip, and tip pinch grip. These findings highlight the task-specific nature of muscular strength among workers in the handloom sector. Additionally, the muscular strength was found to be significantly affected by both years of experience and age, with both factors showing a negative correlation, indicating that as the weavers age or gain more years of experience, their muscular strength tends to decline (45).

#### 3.2.4 Respiratory system

The prevalence of respiratory problems among weavers was 72.73%. It ranked as the second highest next to musculoskeletal disorder. An analysis of gender-wise prevalence of respiratory problems reflects higher prevalence among males (76.92%) than females (66.67%). Moreover, peak expiratory flow rate was used to determine the respiratory-related challenges faced by human beings. The units of measurement of peak expiratory flow rate were liters per minute (L/min) (27). The mean observed peak expiratory flow rate among weavers varied from 460 among weavers aged below 25 years to 357.89 among weavers who were aged above 60 years. However, for non-weavers, the mean observed peak expiratory flow rate ranged from 500 among those aged below 25 years to 403.53 among those above 60 years. A significantly lower expiratory flow rate has been observed among weavers compared to non-weavers across all age groups. Further, the trends of peak expiratory flow rate for weavers have been found to be consistently declining after the age of 45 years. Furthermore, a higher mean peak expiratory flow rate was observed among weavers who had no prevalence of respiratory-related diseases (411.09) than among weavers who had the prevalence of respiratory-related diseases. For example, the lowest mean observed peak expiratory flow rate was found for weavers who had previously experienced pulmonary tuberculosis symptoms (281.49), followed by prevalence of current pulmonary tuberculosis symptoms (302.22), and chronic bronchitis (315.83) (8).

#### 3.2.5 Visual system

Visual impairment consists of blindness and moderate visual impairment. The prevalence of visual impairment among weavers in India was found to be 14 percent, consisting of moderate visual impairment (9.4%) and blindness (4.6%). Visual impairment was said to be rising with increase in age, the highest among those who were 70 years old and above (42.1%), followed by those of 60–69 years (23%) and 50–59 years (10.3%). Educational status was also a significant determinant of visual impairment. Among the weavers, those with formal education a lower prevalence of visual impairment (9.9%) than those with no formal education (18%). Gender was another factor. The prevalence of visual impairment among female weavers (15.6%) was higher than among male weavers (13.1%) (9). Khan and Qayum (27) also supported the gender difference in the prevalence of eye problems, pointing to it being 53.85 percent and 54.55 percent for males and females, respectively.

However, Nag et al. (10) observed a contradictory result of a higher prevalence of the eye problem among male handloom weavers (55.9%) as compared to the female handloom weavers (10.1%). As educational level and age (9) were positively related with the visual system, the possibility of variation in the pattern of gender difference in educational level, distribution of working handloom workers, and years of working might lead to a contradictory finding of the Nag et al. (10) study compared to other studies (9, 27). Also, the variation in the gender difference in the prevalence of the visual system in India across studies is due to methodological differences and geographical variation explained by another study (9).

#### 3.2.6 Other diseases

Quite a few studies found the prevalence of hearing and skin problems among weavers (10, 27). The occurrence of overall skin problems ranged from 68.18 percent to 16.72 percent among them. The findings on the gender difference in the prevalence of skin problems were contradictory. Khan and Qayum (27) found a higher prevalence of skin problems among male weavers (69.23%) than female weavers (66.67%). However, Nag et al. (10) reported a higher prevalence of skin problems among female weavers (22.3%) compared to male weavers (10.8%). The prevalence of ear problems varied from 2.39 percent (10) to 45.45 percent (27). Both the studies found a higher risk of the prevalence of hearing problems for males than females (10, 27).

#### 3.2.7 Occupational risk factors

The reason for the highest prevalence of the musculoskeletal disorder compared to other diseases was the nature of work in weaving. Chantaramanee et al. (46) estimated the risk associated with weaving activity by using the rapid upper limb assessment (RULA) score. This score ranges from 1 to 7 among people in general, a score of 1 indicating the lowest occupational risk and score of 7 indicating the highest occupational risk. The present study found the RULA score of 6.80 among weavers, suggesting the prevalence of a higher occupational risk for the occurrence of musculoskeletal disorders, pointing to the need to take immediate measures for improving the working environment in weaving to minimize the prevalence of such disorders. Bori and Bhattacharyya (25) also used the RULA score and strain index for quantifying the occupational risk factors among the weaving community. This study measured the activity-wise RULA score for weaving, such as weaving (6.41), followed by wrapping (6), and spinning (5.90). The RULA score was found to be higher than 4, which indicated a need for investigation and necessary modification in the working environment.

A strain index scores below 3 implies safe working conditions, 3 to 5 indicates uncertainty, 5 to 7 suggests some risk, and above 7 indicates a high-risk working environment. In weaving activities, strain index score for the right hand ranged from 4.30 in wrapping to 46.59 in weaving. A similar pattern of strain index was also found for the left hand, with the index score ranging from 2.95 in wrapping to 34.81 in weaving. Across all types of weaving activities, there is a significantly higher strain index for the right hand than for the left hand, which suggests a higher risk of the development of musculoskeletal disorders in the right than the left hand (25).

Applying the RULA score, the occupational risk level in weaving activity has been classified into four categories, viz., the RULA scores 1 to 2, 3 to 4, 5 to 6, and 7 were categorized as risk levels 1, 2, 3, and 4, respectively. The study revealed that the highest number of weavers were exposed to risk level 4 (50%), followed by risk level 2 (35.29%), and risk level 3 (14.7%). The risk factors for the prevalence of MSDs were also identified by using the RULA score. The duration of weaving activity was found to be a significant risk factor for the prevalence of musculoskeletal diseases. A higher number of weavers were observed to have been exposed to the risk level 4 among those working more than 8 h a day (35.29%) compared to those working for less than 8 h (20.59%). It is worth mentioning that most of the studies were confined to occupational risk at the workplace in weaving activity, and there is a lack of studies investigating the impacts of chemicals used in dyeing on human health in the handloom sector (43). Islam et al. (47) was the lone study to examine the chemical concentration in handloom dyeing in Bangladesh, which reported a hazardous level of copper, iron, chromium, manganese, lead, and zinc in the dyeing effluent.

### 3.3 Meta-analysis

**Figure 3:**
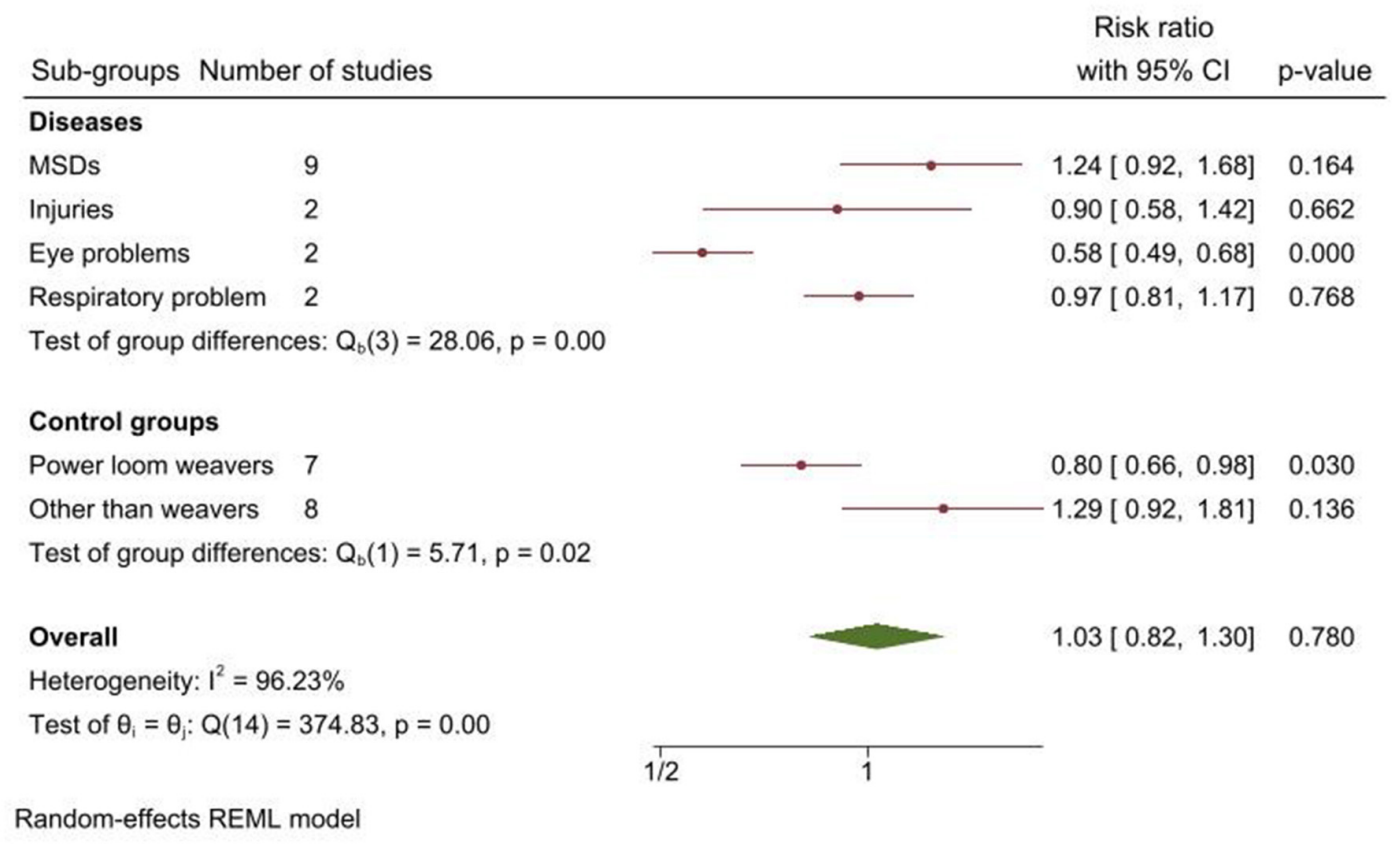
Meta-analysis of health outcomes among handloom weavers compared to control groups. Source: Authors' estimated. MSDs, Musculoskeletal diseases; CI, confidence interval.

**Figure 4:**
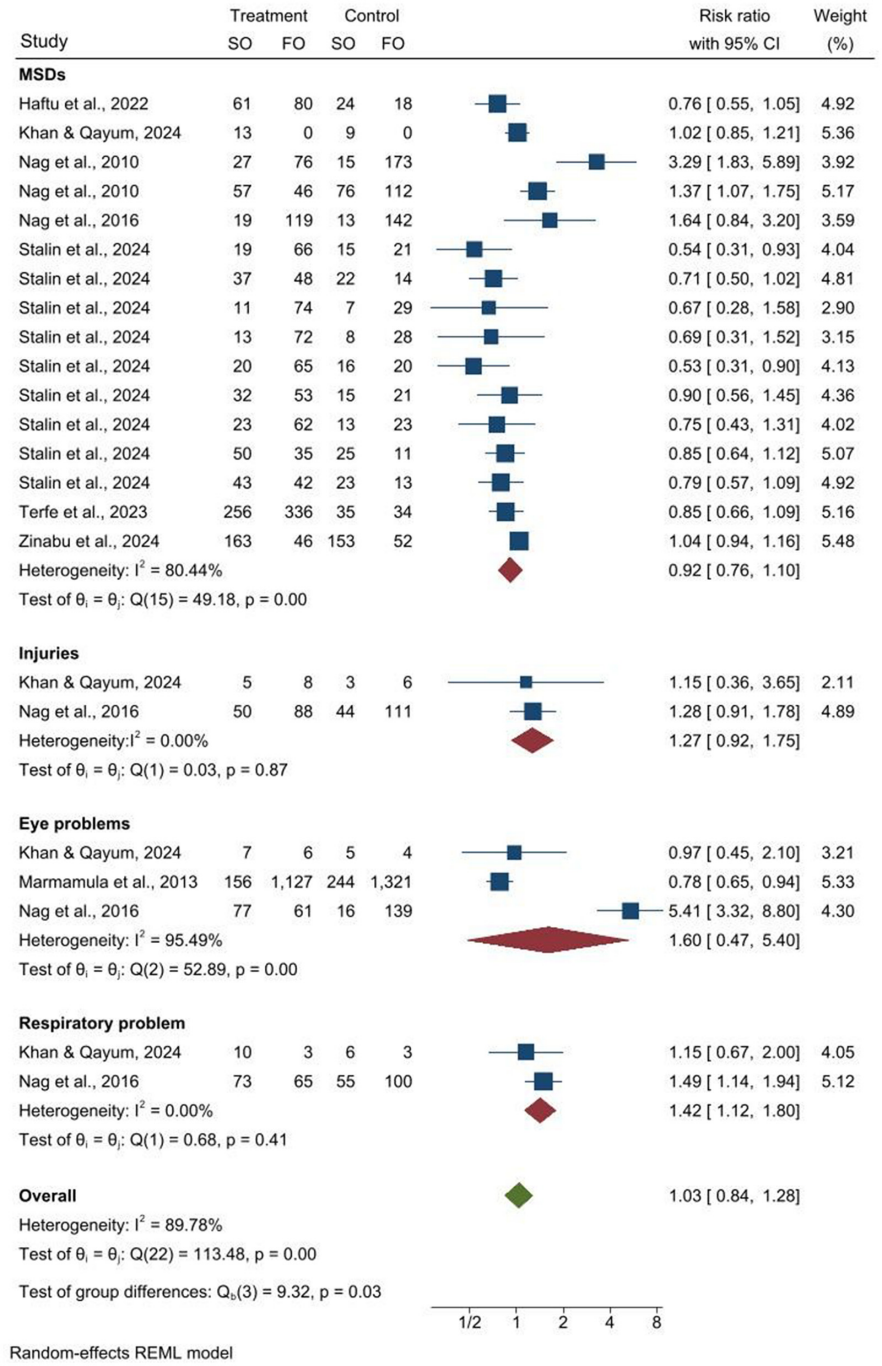
Meta-analysis of gender differences in health outcomes among handloom male weavers (treated group) compared to female weavers (control group). Source: Authors estimated. Treatment group, male; control group, female; SO, Success outcomes; FO, Failure outcomes; MSDs, Musculoskeletal diseases; CI, confidence interval.

The results of the meta-analysis are shown in Figures 3 and 4. In Figure 3, a comparison of health outcomes among handloom workers and the control group has been presented. Drawing on available literature, the meta-analysis focused on four diseases: eye problems, musculoskeletal diseases, respiratory problems, and injuries. A significant (*P*-*value* = 0.00) difference in the risk of the prevalence of diseases among handloom workers compared to the control groups was observed. The prevalence of eye problems among handloom workers was significantly lower than in the control group. The risk ratio for the prevalence of eye problems among handloom workers was 0.58, indicating a 0.42 times (1–0.58 = 0.42) lower risk of the prevalence of the disease among handloom workers in comparison to the control group, which was statistically significant at 1 percent level (*P-value* = 0.00). Similarly, there was lower risk of prevalence of respiratory problems (relative risk = 0.97) and injuries (relative risk = 0.90) among handloom workers than in the control group. However, for musculoskeletal diseases, the relative risk (1.24) of prevalence among handloom workers was higher than in the control group, and it was not significant (*P-value* = 0.164).

The analysis of the relative risk for the prevalence of diseases among handloom workers compared to the different control groups, such as other than weavers, and power loom weavers has been presented in Figure 3. The relative risk for the prevalence of diseases among handloom workers compared to the different control groups was significantly different at 5 percent level (*P*-*value* = 0.02). There was a higher relative risk (1.29) of the prevalence of diseases among handloom worker compared to other than handloom worker. It indicated 0.29 (1.29–1 = 0.29) times higher relative risk of the prevalence of diseases for handloom workers as compared to other than handloom worker. Additionally, the meta-analysis revealed lower relative risk (0.80) of prevalence of diseases among handloom worker in comparison with the power loom worker. So far as overall prevalence of diseases was concerned, the relative risk was found to be 1.03, which implies only 0.03 times higher risk of prevalence of the diseases among handloom weavers than among control groups (*P-value* = 0.780).

The meta-analysis of the prevalence of diseases among handloom male workers compared to female workers is presented in Figure 4. The test for the difference in the prevalence of diseases between male and female handloom workers was found to be significant at 5 percent level (*P-value* = 0.03). For male handloom workers, a significant *P-value* (0.00) showed a higher relative risk (1.42) of the prevalence of respiratory problems than for the female handloom workers.

**Figure 5:**
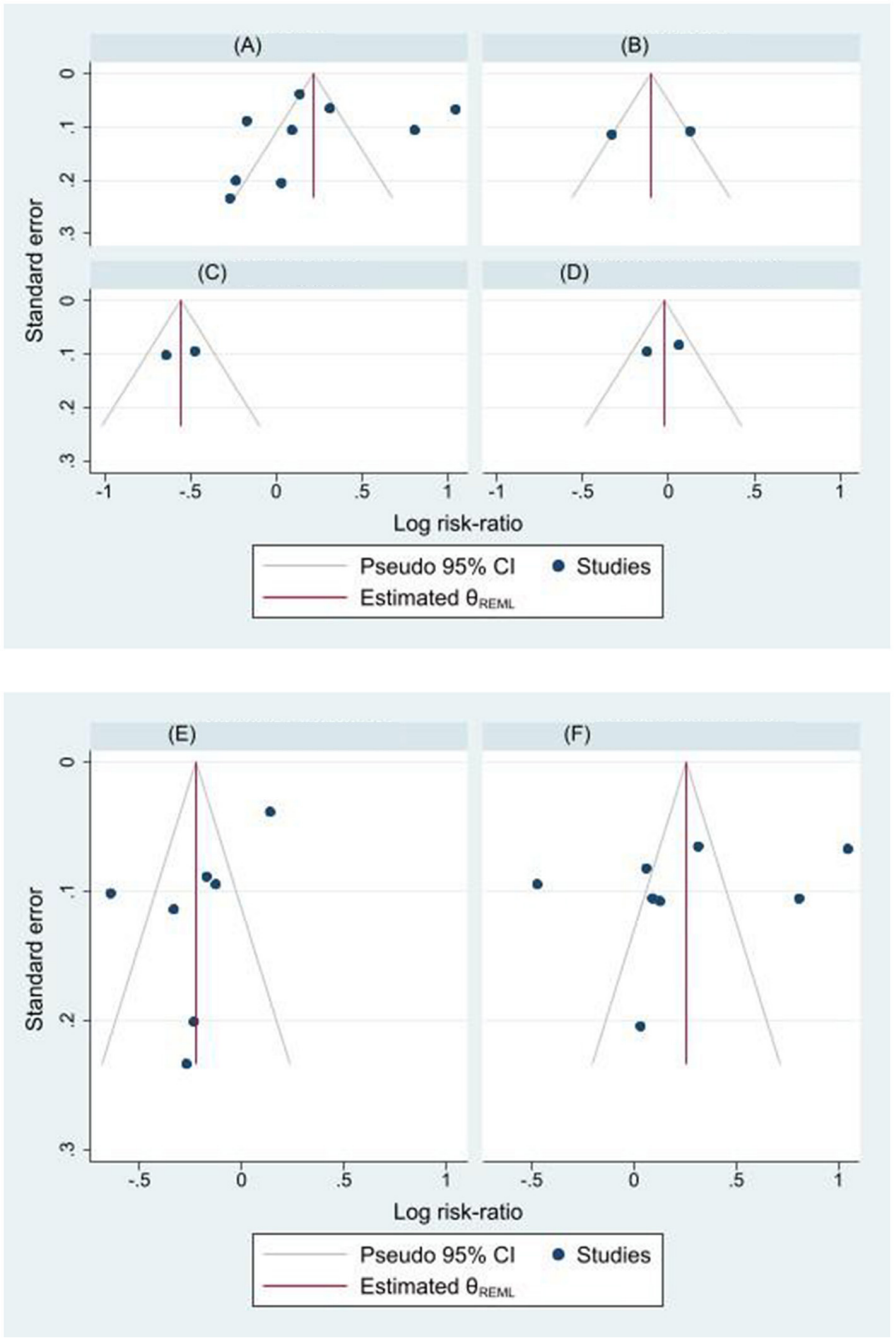
Funnel plot of health outcomes among handloom weavers compared to control groups. **(A)** MSDs, **(B)** injuries, **(C)** eye problems, **(D)** respiratory problem, **(E)** powerloom weavers, **(F)** other than weavers. Source: Authors estimated.

From Figure 5A, it was observed that there was the presence of publication bias for musculoskeletal diseases, and there was no presence of publication bias for diseases such as injuries, eye problems, and respiratory problems as represented in Figures 5B–D, respectively. The Egger's test was used to find out the publication bias for four types of diseases combined. The Egger's test (*P-value* = 0.09) shows there is no publication bias for the comparison of prevalence of diseases among the treated group and the control group.

Figures 5E and F show the presence of the publication bias to compare the prevalence of diseases among handloom workers with the power loom weavers and the others than the weavers due to their asymmetric distribution. However, the Egger's test (*P-value* = 0.34) reflects there was no publication bias to compare the prevalence of diseases among handloom workers with the power loom weavers and the others than the weavers. The Egger's test is preferred over the funnel plot. So, publication bias is determined by using Egger's test.

**Figure 6:**
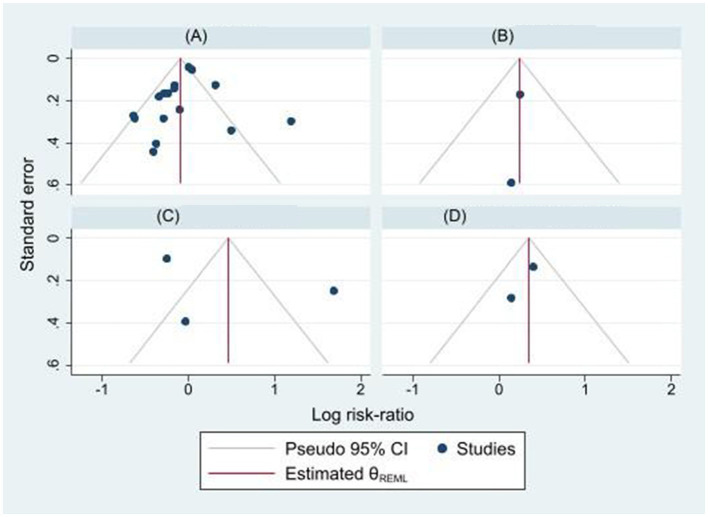
Funnel plot of health outcomes among handloom male weavers compared to handloom female weavers. **(A)** MSDs, **(B)** injuries, **(C)** eye problems, **(D)** respiratory problem. Source: Authors estimated.

In Figure 6A and C are asymmetrically distributed. So, the occurrence of the publication bias for the MSDs and eye problems. In the case of Figures 6B, D, the effect size of studies was symmetrically distributed. Therefore, no occurrence of the publication bias for injuries and respiratory problems. The overall publication bias for comparing the health outcomes among handloom male weavers with handloom female weavers was tested by Egger's test. This test (0.94) shows no presence of publication bias.

Sensitive analysis is presented in Supplementary Figure 8 and 9 shows that there is no significant impact of individual study on the overall effect size for both the cases of health outcomes among handloom weavers compared to control groups and handloom male weavers compared to handloom female weavers, respectively. In sensitive analysis, overall effect size varies from 0.96 (0.79–1.16) to 1.09 (0.87–1.36) for health outcomes among handloom weavers compared to control groups, while for health outcomes among handloom male weavers compared to handloom female weavers varies from 0.96 (0.83–1.12) to 1.06 (0.86–1.32).

## 4 Discussion

The study offers a novel systematic review that employs a bibliometric and meta-analysis to assess the prevalence of the diseases among handloom weavers. The strength of this study lies in the disease-wise and control group-wise estimated relative risk of disease prevalence among weavers compared to other occupations. It provides a clear idea about diseases and a higher relative risk of prevalence among weavers compared to control groups. This study also estimates the overall relative risk of prevalence of diseases among weavers compared to other occupations. It has not only compared the prevalence of diseases between weavers and other occupations but also analyzed gender difference in the prevalence of diseases in the handloom sector.

This study found that MSDs have the highest prevalence among handloom weavers (25–27, 35, 38), and most of the studies were concentrated on these diseases. Meta-analysis estimated the highest relative risk [1.24 (95% confidence interval: 0.92–1.68)] of the prevalence of MSDs among weavers than the control group. Similar observations were reported in some of the previous studies (25, 27, 35). The factors affecting the prevalence of MSDs among weavers were years of experience (17, 23), weaving as a primary occupation, age of weavers (17), long times of sitting and repetitive work (2–5, 7, 23, 42, 45), awkward position (2–5, 7), no adjustments in looms (10), and lack of exercise (2).

The high prevalence of musculoskeletal disease is associated with the rise in the years of working experience and the age of weavers. The prevalence of MSDs among weavers varies from 81.9 percent among those with over 5 years of work experience to 74.6 percent among those with less than 5 years (4). Additionally, Nag et al. (10) reported the highest percentage of handloom workers aged above 35 years compared to the other informal occupations like transportation and fish processors. This is one of the reasons for the higher prevalence of musculoskeletal disorders among weavers, since usually there is a rise in the prevalence of musculoskeletal disorders with age (17, 23, 31).

Most of the handloom weavers are dissatisfied with the loom technique and opine that inflexibility in the handloom technique is the main reason for the prevalence of musculoskeletal disorders. Therefore, remedial measures should include redesigning of weaving instruments to enhance the comfort of weavers as well as scheduled rest during the weaving to reduce physical strain (33). Moreover, the implementation of the new technique in place of the traditional technique could help in minimizing the risk of the prevalence of musculoskeletal diseases. For example, studies found a fall in MSDs from 87.5 percent to 10 percent (48) for weavers after 6 months of working with the new comfortable design in handlooms. Weavers also need to exercise, which helps in prevention of MSDs (3, 30) among them. Generating awareness among handloom weavers about the ways to prevent musculoskeletal disorder among weavers would be helpful too (49).

There is a risk of the prevalence of respiratory problems among weavers largely due to their exposure to dust during yarn preparation and the weaving process (8). Another contributing factor was the lack of proper ventilation in the workplace (50, 51). To prevent this, the Government of India has been providing financial support to weavers for the construction of work-shed exclusively for weaving. This scheme has aided in improving the ventilation in the work-sheds that are dedicated for weaving activities. Visual impairment is yet another prevalent health risk in the handloom sector. The high level of concentration required in weaving is a significant contributor to these issues. The two other leading causes of visual impairment among weavers were prevalence of refractive error (56%) and cataract (33%) (9). Therefore, it is essential to provide healthcare services focused on eye care for weavers that would help in early detection and necessary intervention to mitigate visual impairment.

Meta-analysis shows that the risk of prevalence of diseases among handloom weavers was higher in comparison to all control groups except for power loom weavers. The reasons for the higher prevalence of MSDs among power loom weavers compared to handloom weavers were their double work shifts, working in a standing position, and continuous exposure to vibration (10, 52). The inequality in the prevalence of diseases in the handloom sector was found both across occupational categories and gender. Overall, the male handloom weavers exhibit a higher prevalence of diseases than female handloom weavers. However, the meta-analysis revealed that MSDs have a higher prevalence among females than males and this is consistent with the findings of a previous study (29). Low level of education is identified as a risk factor for the prevalence of MSDs (17). Educational qualification among female weavers is lower than that among male weavers in the handloom sector. It limits females' bargaining power in deciding wages and increases their vulnerability to exploitation by employers. This power imbalance often led to psychological distress among female weavers (52). There are studies that found a positive correlation between psychological disorders and musculoskeletal disorders (4, 23). This explains the higher prevalence of musculoskeletal disorders among female handloom weavers. The higher physical workload associated with housekeeping work also contributes to the higher prevalence of musculoskeletal disorders among female handloom weavers (50).

There is comparison made for the health risk among handloom workers with other occupation like small scale construction workers, refuse collectors, and Harare beverage company workers. The similar types of risk of prevalence of the MSDs are found between handloom workers and construction workers due to both occupations are physically demanding occupation (53). The awkward posture among refuse collectors also leads to the higher prevalence of MSDs among refuse collectors (54) as like among the handloom workers. So, there is a need for training among handloom workers by collaborating the different stakeholder to minimize the risk of occupational health hazards. Moreover, from a safety point of view a safe workplace like Harare beverage company workers can reduce the injury among handloom workers (55), as the study noted there is a higher occurrence of occupational injuries seen among the male counterpart.

There is no presence of publication bias in the present study. However, the presence of a higher heterogeneity issue, which is a limitation of this study despite this, is the first systematic review and meta-analysis on the health burden of diseases among handloom weavers. A leave-one-out meta-analysis was conducted to identify the cause of heterogeneity. The leave-one-out analysis found that there is no significant impact of individual study on the overall effect size. Therefore, the possible sources of heterogeneity are multiple diseases, different control groups, and various locations included in the pooled analysis (56). Another limitation is the present study could not validate these findings by using the primary data, as this study is based on the data compiled from the review of literature. Therefore, further research should focus on the primary study to validate these findings.

## 5 Conclusions

A higher risk of musculoskeletal disorders has been observed among handloom weavers with a variation in the prevalence of specific diseases between handloom weavers and the control group. The other occupational related health issues identified among handloom workers include eye strains, respiratory illnesses, digestive disorders, and visual impairments. There is also significant variation in the prevalence of specific diseases was observed between male and female weavers in the handloom sector.

Methodologically, most of the studies relied on the traditional techniques, primarily focusing on the prevalence of diseases for assessing occupational health hazards among the handloom weavers. However, the incidence of diseases and mortality therefrom related to occupational hazards in weaving activity has remained unexplored. Future research should incorporate modern techniques like Disability-Adjusted Life Years (DALYs) and Quality-Adjusted Life Years (QALYs) to assess comprehensively the burden of disease and quality of life of the handloom weavers. The strength of this study lies in its attempt to focus on the prevalence of all diseases among handloom weavers and meta-analysis. However, one limitation of the present analysis is that a relatively small number of studies were included in the meta-analysis due to the fact that the number of studies comparing the prevalence of diseases among handloom weavers with a control group was also small. Future research needs to focus on the comparison of the prevalence of diseases between handloom weavers and control groups.

## Data Availability

The original contributions presented in the study are included in the article/Supplementary material, further inquiries can be directed to the corresponding author.
