# Supplementary material for: Occupational health burden of handloom workers: a systematic review and meta-analysis
Source: Front Public Health. 2026 Jul 22;14:1828924. doi: 10.3389/fpubh.2026.1828924 (PMC13440152; doi:10.3389/fpubh.2026.1828924)
Supplement: Supplementary file 1 [file Data_Sheet_1.docx]

**Supplementary Materials**

**Search Strategy for Scopus**

ALL(health hazard) OR ALL(occupational hazard) OR ALL(occupational health) OR ALL(burden of disease) OR ALL(burden of health) OR ALL(musculoskeletal diseases) OR ALL(work stressors) OR ALL(back pain) OR ALL(blindness) OR ALL(vision loss) OR ALL(glaucoma) OR ALL(age-related macular degeneration) OR ALL(refraction disorders) OR ALL(other vision loss) OR ALL(prevalence) OR ALL(incidence) OR ALL(morbidity) OR ALL(mortality) OR ALL(epidemiology) AND ALL(handloom)

**Bibliometric Analysis**

Supplementary Table 1 presented number of documents were published on occupational hazard in the handloom sector. The first document on the health problems of handloom weaving was published in 1980. Between 1980 and the end of February 2025, a total of 554 documents were published with an annual growth rate of 1.55%. This growth rate shows that earlier, this area of research was relatively neglected by researchers. An analysis of the documents published in the domain of occupational health in the handloom sector found that 57.58% were articles, followed by books (20.40%), review articles (8.48%), and book chapters (7.95%).

**Table 1** Information about documents on occupational hazards in the handloom sector

| Description | Results (Percentage in parentheses) |
| --- | --- |
| Timespan | 1980-2025 |
| Documents | 554 |
| Annual growth rate % | 1.55 |
| Document types | |
| Article | 319 (57.58) |
| Book | 113 (20.40) |
| Book chapter | 44 (7.95) |
| Conference paper | 28 (5.05) |
| Note | 1 (0.18) |
| Review article | 47 (8.48) |
| Review book | 1 (0.18) |
| Review book chapter | 1 (0.18) |
| Total | 554 (100) |

Source: Authors calculated

Trends in the number of documents published annually on occupational health issues in the handloom sector have been presented in Supplementary Figure 1. The frequency of the publication of documents per year represents the focus devoted to a specific field. From 1980 to 2002, the publication remained stagnant, indicating limited academic interest in this field. A gradual rise in the annual production of documents occurred between the period 2003 and 2017, followed by a steady rise after 2017, except in 2020, likely due to the COVID-19 pandemic. These trends in the annual publication of documents on the occupational health of workers in the handloom sector reveal that it was overlooked until 2011 and gained increased attention among researchers thereafter.

**Figure 1** Trends in the annual number of document production

 Source: Authors calculated

Supplementary Figure 2 presents the top ten prolific authors in the area based on the total number of documents published. Meena, M. L. had the highest contribution to the issues of occupational health among handloom weavers, who published 11 documents, followed by Singh, A. K. with 10, and Dangayach, G. S. with 8 documents. All authors listed under the top ten authors published at least four documents related to occupational hazards among handloom workers.

**Figure 2** Top ten authors in the field of occupational hazards in the handloom sector

 Source: Authors calculated

Keywords were used to search the relevant documents in the specific domain. The present study identified the top fifty keywords from the published literature. Keyword-wise, the highest number of published documents has been identified as ergonomics (33), followed by India (17), entrepreneurship (16), handloom (15), COVID-19 (13), musculoskeletal disorders (13), handloom weavers (10), sustainability (10), musculoskeletal disorder (9), and low back pain (8), with the number of documents given in parentheses. The used keywords indicate that most of the studies primarily focused on ergonomic aspects and the prevalence of musculoskeletal disorders, particularly low back pain among handloom workers. However, the less frequently used keywords were occupational health (5), safety (4), and health (4). It indicates that a smaller number of studies have attempted to identify the safety practices among handloom workers at the workplace, highlighting a significant research gap in the field of occupational health burden in the handloom sector (Supplementary Figure 3).

**Figure 3** Tree map of the top 50 keywords relating to occupational hazards in handloom

 Source: Authors’ calculated

Supplementary Figure 4 shows the top ten most relevant institutions that published the highest cumulative number of publications between 1980 and 1925. The number of publications affiliated with each institution represents the research potential of a specific institution on health issues in the handloom sector. The Malaviya Institute of Technology led with 11, followed by the Indian Institute of Technology Kharagpur (8) and the National Institute of Fashion Technology (7). Among the top ten institutions, the Indian Institute of Technology Kharagpur, National Institute of Fashion Technology, Banaras Hindu University, and Jawaharlal Nehru University were prominent names from India. This highlights the significant contribution of Indian institutions focusing on the health-related issues faced by handloom workers.

**Figure 4** Top ten most relevant affiliations

 Source: Authors’ calculated

Supplementary Figure 5 represents the top ten countries based on the cumulative number of publications between 1980 and the end of February 2025. As revealed by the data, India has been dominating in the publications on occupational health problems in the handloom sector. The share of India’s publications in the total number of 554 documents published was 326, or 58.84 percent. The United States of America (110) and the United Kingdom (94) occupy second and third positions, respectively, out of a total of 554 documents published. It was pertinent to note that the summation of country-wise publication counts might exceed 554 documents due to the multi-country research yielding one or more documents.

**Figure 5** Top ten countries in the number of documents produced

Source: Authors’ calculated

| Countries | Number of documents |
| --- | --- |
| INDIA | 326 |
| USA | 110 |
| UK | 94 |
| CHINA | 61 |
| BANGLADESH | 39 |
| AUSTRALIA | 38 |
| IRAN | 34 |
| INDONESIA | 28 |
| CANADA | 23 |
| ETHIOPIA | 21 |

Trending topics in the handloom sector represent the changes in the priority area of research over time. In this paper, the trending topic in terms of title was considered by taking two words of a title, and the results are presented in Supplementary Figure 6. It may be seen that the most frequently worked and current trending topics were cross-sectional study, work-related musculoskeletal disorder, and human health. Over the period of time, it is found that musculoskeletal disorder was the most trending topic represented by the highest term frequency (25), followed by ergonomic evaluation (20).

**Figure 6** Trend topics by title (bigram)

Source: Authors’ calculated

To know the changes in the theme of research on occupational health issues in the handloom sector, a thematic evolution analysis was done for the period between 1980 and 2025, as shown in Supplementary Figure 7. It was evident that during 1980-2000, research largely focused on the industrial revolution in the handloom sector. In the succeeding decade (2001–2010), a shift occurred with the emergence of repetitive strain as a new focus area of research, highlighting the physical strain on handloom workers caused by repeated weaving and related tasks. In the past decade (2011-2020), musculoskeletal disorders, occupational health, and anthropometric data were added to handloom-related health research. After 2020, research themes further diversified to ergonomic evaluation, human health, dietary diversity, and life cycle issues, which provide a holistic approach to studying occupational health in the handloom sector.

**Figure 7** Thematic evolution in the occupational hazards in the handloom sector

 Source: Authors’ calculated

**Leave-one-out sensitivity analysis**

**Supplementary Figure 8** Sensitive analysis of health outcomes among handloom weavers compared to control groups

Note: MSDs= Musculoskeletal diseases, EP= Eye problems, RP= Respiratory problems

**Supplementary Figure 9** Sensitive analysis of gender differences in health outcomes among handloom male weavers (treated group) compared to female weavers (control group)

Note: MSDs= Musculoskeletal diseases, EP= Eye problems, RP= Respiratory problems
